# OCCUPATIONAL MOBILITY AND CHRONIC ILLNESS IN LATE LIFE: A SYSTEMATIC REVIEW

**DOI:** 10.1093/geroni/igz038.1180

**Published:** 2019-11-08

**Authors:** Rong Fu, Kathleen Abrahamson, Tara Campbell

**Affiliations:** 1 Siena College, Loudonville, New York, United States; 2 Purdue University, West Lafayette, Indiana, United States

## Abstract

Objectives: Occupational mobility at various stages in the life course may have a cumulative influence on health outcomes in later life. This study aims to (1) systematically review the association between occupational mobility and chronic illness in late life; and (2) identify potential mechanisms underlying this relationship. Methods: A systematic review of literature was carried out by searching two databases (PubMed and SocIndex) and reference lists. Eligible studies examined associations between occupational mobility and at least one measure of chronic illness among adults aged 50 years or above. Occupational disruptions (e.g., job loss) were reviewed as special cases of occupational mobility. Results: Downward occupational mobility and mid-life occupational disruptions have been consistently shown to predict higher risk of chronic illness in older adults. Several potential mechanisms were identified from the literature: (1) health behaviors, including dietary practices and alcohol consumption; (2) psychosocial factors, including stress, stigma, job control, job demands, and job satisfaction; (3) economic factors, including financial incentives or constraints; and (4) other individual characteristics, including personality traits and coping skills. There is also evidence that the timing of job mobility and the duration of (each) occupation modify the association between occupational mobility and health in late life. Discussion: These findings suggest that experiencing involuntary occupational mobility at various stages in the life course can increase the risk of chronic illness in late life. Health professionals and policymakers should target more resources to disadvantaged older adults who experience involuntary occupational transition.

